# BEASTling: A software tool for linguistic phylogenetics using BEAST 2

**DOI:** 10.1371/journal.pone.0180908

**Published:** 2017-08-10

**Authors:** Luke Maurits, Robert Forkel, Gereon A. Kaiping, Quentin D. Atkinson

**Affiliations:** 1 School of Psychology, University of Auckland, Auckland, New Zealand; 2 Department of Linguistic and Cultural Evolution, Max Planck Institute for the Science of Human History, Jena, Germany; 3 Leiden University Centre for Linguistics, Leiden University, Leiden, the Netherlands; Estonian Biocentre, ESTONIA

## Abstract

We present a new open source software tool called BEASTling, designed to simplify the preparation of Bayesian phylogenetic analyses of linguistic data using the BEAST 2 platform. BEASTling transforms comparatively short and human-readable configuration files into the XML files used by BEAST to specify analyses. By taking advantage of Creative Commons-licensed data from the Glottolog language catalog, BEASTling allows the user to conveniently filter datasets using names for recognised language families, to impose monophyly constraints so that inferred language trees are backward compatible with Glottolog classifications, or to assign geographic location data to languages for phylogeographic analyses. Support for the emerging cross-linguistic linked data format (CLDF) permits easy incorporation of data published in cross-linguistic linked databases into analyses. BEASTling is intended to make the power of Bayesian analysis more accessible to historical linguists without strong programming backgrounds, in the hopes of encouraging communication and collaboration between those developing computational models of language evolution (who are typically not linguists) and relevant domain experts.

## Introduction

Recent years have seen an increased interest in the use of computational and especially Bayesian methods for inferring phylogenetic trees of languages within an explicit, model-based framework [1–13]. The recency of this trend means there is currently a lack of software tailored to the needs of this sort of analysis of linguistic data. Thus, published analyses to date have all relied on software developed for biological phylogenetics, such as BayesPhylogenies [14], BEAST [15, 16] or MrBayes [17, 18].

Amongst these existing pieces of software, BEAST 2 is unique in that it was deliberately designed from the ground up to support user extensibility. It is possible for users to write packages which extend the phylogenetic modelling capabilities of BEAST 2, by e.g. specifying new tree priors or new substitution models. This makes BEAST 2 an appealing platform for the burgeoning field of computational linguistic phylogenetics, as those working in the field have the ability to develop language-specific models and readily share them amongst themselves, in a process requiring no input from the BEAST 2 development team.

In this paper we provide a brief overview of the application of computational phylogenetics to lingustics and introduce a new software tool named *BEASTling*, which provides an interface to BEAST 2 designed specifically for linguistic applications. The goal of BEASTling is to make the specification of BEAST analyses for linguistic phylogenetics simple and accessible, even when the analyses are large or complicated, and to encourage the reproduction, validation and extension of published analyses. By making the power of BEAST accessible to a broader range of interested researchers in linguistics, we hope to foster increased collaboration between experts in linguistics and computational evolution to further and faster develop a formal, quantitative and data-driven approach to historical linguistics.

## Bayesian linguistic phylogenetics

The recent explosion of interest in linguistic phylogenetics has quickly settled on a standard framework of Bayesian inference using probabilistic models of language evolution. In this framework, languages are placed into a binary phylogenetic tree and linguistic data, such as cognacy judgements or structural/typological observations, are associated with the leaf nodes of the tree, representing extant or recently extinct languages. The data is assumed to have been generated via a probabilistic process defined on the tree, with features taking a particular value at the root and then potentially changing along each branch. The probability of a feature changing value depends upon the length of the branch. The model for calculating probabilities can be simple or quite complicated, with multiple parameters controlling the behaviour.

Bayesian inference is performed using Markov Chain Monte Carlo (MCMC). In this procedure, the phylogenetic tree and other model parameters are typically initialised to random starting values. Then, both the tree and the parameters are subject to a long series of small, random changes, e.g. part of a tree may be “pruned” off and then “regrafted” elsewhere, or the length of a branch may be scaled by a random factor. If one of these randomly proposed changes increases the likelihood of the data, it is accepted, whereas if the likelihood is decreased the change may be rejected with some probability proportional to the decrease. At regular intervals, the tree and all model parameters are sampled until a large number—say 10,000—of sampled trees has been collected. The probabilities of accepting or rejecting proposals are calculated in such a way that the resulting sample of trees represent independent samples from a posterior distribution, which uses Bayes’ theorem to combine the likelihood of the observed data under the chosen model with a prior distribution which represents *a priori* expectations about the tree.

One of the strengths of this framework is that the prior probability distribution can be used to include existing linguistic knowledge in an analysis. For example, tree topologies which violate some known truth—that a particular set of languages are related—can be given a prior probability of zero, in which case they will never be sampled. Or, if the approximate age of a common ancestor of some languages is known, say due to the dating of written artifacts, then a prior distribution can ensure that trees are increasingly less likely to be sampled as the age they assign that ancestor deviates further from the provided estimate. Any aspect of the model may be constrained via the use of prior distributions if the researcher believes that such a restriction is well-supported by previous research.

Another strength is that the output is not a single tree or a single set of parameter values, but rather a large collection of sampled trees and values. The variation across the trees in the posterior sample conveys information about how certain the linguistic data suggests we should be about certain details. Two languages may be siblings in 900 out of 1,000 sampled trees if the data strongly supports their being related, while two other languages may be siblings in 500 trees but unrelated in 500, suggesting that the data is inconclusive on this point. Just as any aspect of the model may be constrained by the prior, every aspect of the model also automatically comes with a direct measure of probabilistic uncertainty.

This framework is widely applicable to problems of interest in historical linguistics. Things which can be estimated, complete with measures of uncertainty, include the internal structure of family trees, the time depth of the root of the tree or the most recent common ancestor of any set of languages, the geographic location of protolanguages, and the amount of variation in the rate of change across features in dataset (e.g. across meaning slots in cognate data or across different features in a typological survey), as well as providing an explicit ranking of features by rate. Prior distributions can be used to ensure that estimates are consistent with accepted or theorised relationships, age estimates and geographical locations. Multiple kinds of prior information can be combined in a single analysis without problems.

## Design and implementation

BEASTling analyses are focussed on the inference of phylogenetic trees from linguistic data. It is also possible for users to provide a known and trusted phylogenetic tree which is held fixed during the analysis, so that model parameters may be estimated conditional on that tree.

BEASTling is not itself a tool for performing MCMC analyses, but rather it is a tool to facilitate configuring an existing MCMC tool, namely BEAST. When using BEAST, analyses are specified using a single eXtensible Markup Language (XML) file, which contain the input data, all the details of the modelling, including tree constraints, substitution models, clock and mutation rate variation, prior distributions for trees and all parameters, as well as the various mechanisms for drawing MCMC proposals, details on which parameters to log, etc. These XML files have a complicated structure which may appear cryptic to new users, and for complex analyses of large data sets the files can easily be thousands of lines long.

BEASTling takes the form of a command-line program written in the Python programming language (Python versions 2.7 and 3.4+ are supported) which transforms its own kind of configuration file into a BEAST XML file, effectively specifying an alternative configuration file format for BEAST. Noteworthy modelling features supported by the current BEASTling release (1.2.1) are Gamma-distributed rate heterogeneity across features, relaxed and random local clocks, age calibrations on clades, monophyly constraints, a range of substitution models suitable for linguistic data and one phylogeographic model, which supports spatial calibrations on clades. Full details of the modelling options available in BEASTling are given in the section “modelling details”.

The format of BEASTling’s configuration files and the process by which they are transformed was designed to satisfy the following design criteria.

### Easy model specification

BEASTling configuration files are designed to be short, neat, human-readable, and intuitively editable by hand. Analyses are specified at a high-level of abstraction, roughly corresponding to the details that a typical user might have in their head when designing an analysis, e.g. “I’d like to infer a phylogenetic tree of the Austronesian languages, using structural data from the World Atlas of Language Structures [19] (WALS) and the Lewis Mk substitution model [20], with a relaxed clock and rate variation across features”. Typical BEASTling configuration files are approximately 10-20 lines long, meaning they can be viewed on-screen in their entirety without scrolling and can easily be included inline in emails or in publications. An example valid configuration file, implementing the Austronesian analysis just described, is shown below. The configuration also includes a calibration on the age of the protolanguage (4,700-5,700 years).

[admin]

basename = austronesian_wals

[MCMC]

chainlength = 50000000

[languages]

families = Austronesian

monophyly = True

[model wals]

data = wals_data.csv

model = mk

rate_variation = True

[clock default]

type = relaxed

[calibration]

root = 4700 - 5700

The syntax of BEASTling configurations files is intended to be expressive: Often only one or two lines need to be added to a configuration in order to include powerful additional modelling components which may correspond to many hundreds of lines of XML. The user is expected to provide only the minimal amount of information required for BEASTling to make sensible high-level decisions about the underlying phylogenetic models in accordance with emerging best practices. This approach necessarily affords the user less control than requiring them to explicitly specify each and every detail of the analysis, however BEASTling attempts to provide sufficient control and flexibility that appropriate models can be specified for most investigations.

### Encouragement of replication

Ideally, scientific software should not only make it easy for the original researcher to tell the computer what to do, but should also make it easy for other researchers to understand the original researcher’s intentions and to use published work as a departure point of work for their own. It is not only important that other researchers be able to exactly replicate a published analysis to confirm the results, but it should also be as easy as possible to make small, precise changes to published analyses in order to investigate the consequences of things such as changing the values of contentious data points or exploring different modelling assumptions. Further, software should encourage “shoulder standing”, so that published analyses can be easily improved and extended with additional data or more realistic models. BEASTling’s clean configuration files serve this purpose: not only are they easy to write, they are easy to read and easy to *re*write. Several additional features are intended to further encourage and facilitate replication.

When a BEASTling configuration file is transformed into a BEAST XML file, the text of the original BEASTling configuration is embedded at the start of the XML file in a comment block, along with the date and time the transformation was performed and the version of BEASTling which was used. Because of the clean, high-level nature of BEASTling configurations, this goes a long way to making the generated XML file self-documenting. The broad nature of an old, forgotten analysis file, or one being subject to peer-review, can be understood at a glance, even if the corresponding XML is thousands of lines long. Furthermore, BEASTling provides an option to embed copies of all referenced data files in the XML file as additional comment blocks. The resulting XML file is then entirely self-contained, allowing researchers to distribute in a single file everything required to precisely reproduce and to modify the analysis in question.

In addition to the XML file, BEASTling can optionally also produce an “executive summary” report (in MarkDown format) detailing the languages and modelling involved in an analysis, and a map of the languages’ locations (in GeoJSON format). Both of these files will be automatically rendered by GitHub if committed to a repository there, encouraging users to share their analyses in this way, which in turn further encourages replication and modification of published analyses.

### Broad data compatibility and intelligent processing

Reflecting its origin in computational biology, phylogenetic software often expects input data to be specified in file formats designed for genetic data, such as the NEXUS file format [21]. Linguists are unlikely to have their data formatted in this way, meaning they are forced to reformat their data prior to analysing it. While no widespread standard file format is yet in use for linguistic data, BEASTling supports two different structures of specifying linguistic data tables, which can be stored in comma separated value (CSV) or tab separated value (TSV) file formats: one is an emerging standard file structure, expecting and encouraging further adoption; the other is a “lowest common denominator” structure, making data provision easy in the interim. Files in the TSV and CSV formats can be easily viewed or edited using a wide variety of programs on any computer platform, including standard spreadsheet applications and existing libraries for most commonplace programming languages.

The first data file structure BEASTling supports is the Cross-Linguistic Data Format [22], a recently introduced format standardizing exchange of data within the Cross-Linguistic Linked Data project and related database projects (see http://cldf.clld.org/ for more on CLDF). This makes it extremely easy to specify analyses using data from existing databases which use the CLDF format, such as WALS, South American Indigenous Language Structures [23] (SAILS) and the electronic World Atlas of Varieties of English [24] (eWAVE). A very similar data structure is used by LingPy [25], a Python library for historical linguistics.

The other is a simple “matrix” structure, in which each row corresponds to one language, and contains that language’s datapoints for every linguistic feature in the dataset, with each column corresponding to one feature.

Regardless of which of these formats is used, BEASTling aims to provide flexibility in the encoding of data, to minimise the amount of work required to use existing data files. As long as the data file conforms to one of the two supported file formats, names for languages and features can be arbitrary strings (though each language and feature must have a unique name). Feature values similarly can be arbitrary strings, with distinct strings being treated as distinct values. Thus, BEASTling will readily accept data files where different feature values are coded numerically (0, 1, 2, … or 1, 2, 3, …), alphabetically (A, B, C, …) or in any consistent freely written form (SOV, SVO, VSO, VOS, OVS, OSV for word order, for example). Missing data points can be represented by question marks (?).

BEASTling can also automatically handle many data-preprocessing chores such as automatically filtering a dataset down to only a specified set of languages and/or features, removing languages which have only missing data for the selected feature set, removing features which have only missing data or constant values for the selected language set, and recoding cognate judgements into independent binary features. It is possible to specify multiple datasets in a single BEASTling analysis, and each dataset may be assigned a different substitution model. This allows combining cognate data and structural data in a single analysis, with appropriate models used for each. If the languages present in multiple datasets do not match exactly, BEASTling can produce analyses using either the intersection or the union of the languages involved. If the union is specified, languages will be treated as having missing data for all features defined in datasets from which they are absent. Automating this sort of data processing not only saves time, but also reduces the likelihood of researchers making minor errors when performing the changes manually, which may go unnoticed.

### Integration of expert knowledge

The recent dawn of computational methods for historical linguistics should not be an impetus for discarding the valuable findings of decades of previous scholarship. Bayesian modelling permits the integration of existing expert knowledge into analyses, and BEASTling is designed to make such integration as easy as possible.

BEASTling features integration with the Glottolog language catalog [26], which “aims to provide a comprehensive list of languoids (families, languages, dialects)”. The 7,748 spoken languages in Glottolog (as of version 2.7) are classified in a principled manner into a collection of phylogenetic trees comprising some 241 families with more than one member. All clades in the Glottolog classification are provided with names (e.g. Standard English belongs to the Macro-English clade, which is a subclade of Mercian, which is a subclade of Anglian, and so on, up through West Germanic, Northwest Germanic and Germanic to Indo-European) as well as with alphanumeric identifiers called “glottocodes” (e.g. Standard English is stan1293). Because the Glottolog classification is made available under a Creative Commons Attribution-ShareAlike 3.0 license, it is possible to include a machine-readable copy of the classification in BEASTling. If users ensure that languages in their datafiles are referred to either by their three letter ISO-639-3 code or by their glottocode, then BEASTling is aware of the Glottolog classification of the languages in the dataset, and this facilitates several useful features.

Most importantly BEASTling is able to impose monophyly constraints on its analyses which enforce consistency with the Glottolog classification. This means that, for example, in a BEASTling analysis involving the Indo-European languages, trees in which the Germanic, Romance, Slavic languages etc. are not appropriately organised into distinct clades will be assigned a prior probability of zero. This ensures that the posterior distribution of trees and model parameters are grounded in a widely recognised and respected pre-existing expert classification.

BEASTling’s Glottolog integration also makes it very convenient to add important details to configurations. Any clade in the Glottolog classification can be referred to by name or glottocode for the purposes of imposing calibration dates or selecting subsets of data files. This makes it quick and convenient to add important pre-existing linguistic knowledge to analyses. For example, BEASTling recognises “Imperial Latin” as a particular node within the Indo-European clade, and we can refer to it by name to use the known age of the Roman empire to provide a calibration date range, rather than having to manually list all of the languages in our analysis which are descended from Imperial Latin.

Glottolog clade names can also be used to specify a subset of languages in a dataset to use for an analysis, enabling the user to easily extract, say, only the Indo-European and Uralic languages from a global database. This removes the need to maintain multiple copies of the same data for use with modelling different sets of language families, once again reducing the chance of minor errors where one file is updated but another is not.

Finally, Glottolog also provides geographic location data for the vast majority of its languages, in the form of latitude and longitude values and an assignment to one of six ‘macroareas’ [27]. BEASTling automatically uses this data to specify leaf node locations for phylogeographic analyses, removing the need for the daunting task of researching and manually enter locations for each language in a large analysis. Alternative location data for families can be easily loaded from a CSV file, overriding the Glottolog data if required. It is also possible to filter datasets by macroarea instead of or as well as by family. Using both filters allows, for example, succinctly selecting all the Austronesian languages in a dataset except for the geographic outlier Malagasy which is spoken in Madagascar.

These features demonstrate the importance and the power of making broadly useful linguistic data available in machine-readable formats under permissive copyright licenses and of referring to languages using standardised, unique identifiers.

Glottolog is an ongoing effort, and names and classifications are subject to change between releases. Each release of BEASTling will be packaged with the latest Glottolog release available at the time (currently 2.7). However, any previous Glottolog release back to 2.4 can be specified in a BEASTling configuration file, and BEASTling will download the appropriate data from glottolog.org. This allows published analyses to be accurately replicated and modified by future releases of BEASTling, even after new Glottolog releases have been made.

### Advanced features

Good software should not only make easy tasks easy, it should also make difficult tasks possible. In addition to functioning as a command-line program for transforming BEASTling configuration files into BEAST analysis specifications, BEASTling can also be used as a library from within Python scripts.

When used in this fashion, it is possible to generate BEAST XML files without first creating a BEASTling configuration file. Instead, the high-level analysis parameters are specified as attributes of a Python object. This is convenient for programmatically generating large numbers of BEAST XML files in which either the same model specification is used for several different datasets, or a range of slightly different model specifications are used for one dataset This is particularly useful for large simulation studies. Quickly generating a large number of BEAST XML files can also be achieved by asking BEASTling to read the linguistic data from stdin, and using shell (e.g. Unix bash) functionality to generate variant input.

When used in this fashion, it is possible to generate BEAST XML files without first creating a BEASTling configuration file. Instead, the high-level analysis parameters are specified as attributes of a Python object. This is convenient for programmatically generating large numbers of BEAST XML files in which either the same model specification is used for several different datasets, or a range of slightly different model specifications are used for one dataset This is particularly useful for large simulation studies. Quickly generating a large number of BEAST XML files can also be achieved by asking BEASTling to read the linguistic data from stdin, and using shell (e.g. Unix bash) functionality to generate variant input.

For more complicated projects, partial analysis specifications can be separated into multiple files. When BEASTling is provided with a list of several files, settings in later files override those in earlier files, similar to multiple inheritance in Object Oriented Programming. This enables many different variations of an analysis to be constructed efficiently simply by including or excluding different files, without duplicating common content across multiple files.

The current BEASTling release supports several different substitution models useful for inference of language phylogenies with linguistically reasonable defaults, as will be described in detail in the following section. In addition, an advanced user can write their own Python class for a model not covered by BEASTling yet and specify it in a BEASTling configuration file, without having to modify BEASTling’s code. This makes BEASTling useful even for advanced users who are developing new models.

## Modelling details

BEASTling analyses use the Yule pure birth process [28] to define a prior distribution over phylogenetic trees. The birthrate parameter is constant over all locations on the tree, but the particular constant value is inferred during the MCMC procedure. The Yule prior is one of two tree prior families supported by BEAST, and in biological applications is typically used to constrain trees over multiple species, i.e. the branching events are interpreted as speciation. The other supported family is the coalescent process [29], which is typically used for trees over populations of a single species, i.e. the branching events are interpreted as reproduction. Coalescent trees have a characteristic shape in which the oldest branching events are very much older than the most recent. There is no theoretical basis for expecting the language diversification process, which is more often analogised to speciation than within-population variation, to yield trees with this shape, nor is there empirical evidence in any established reconstructions. BEASTling therefore prefers the Yule prior. One shortcoming of this approach is that the Yule model assumes that languages never go extinct, when in fact language extinction is believed to be a frequent occurrence. The development of new tree priors specifically designed for linguistic phylogenetics is a continuing area of research, and future releases of BEASTling will include support for any suitable new tree priors implemented for BEAST.

BEASTling supports a number of different clock models, for controlling how tree branch lengths are converted into a measure of evolutionary time; strict clocks, where the same constant rate is applied all over the tree, relaxed clocks [30], where each branch has its own rate sampled from a tree-wide distribution (Lognormal, Exponential and Gamma distributions are supported), and random local clocks [31] which interpolate between these two possibilities. If no calibration dates are provided, then the rate of a strict clock or the mean rate of a relaxed or random local clock is fixed at 1.0, and the branch lengths of the tree can then be interpreted as having units of “expected number of substitutions per feature”. However, if calibration dates are provided for any clades in the tree, then the tree branch lengths are in the same units as the calibration dates, and the appropriate corresponding clock rate is inferred. Date calibrations can be combined with Yule priors exactly in certain cases [32] and only approximately in others. Both approaches are supported by BEAST, and BEASTling automatically applies the exact method when appropriate. When calibration dates are specified, BEASTling will also automatically include appropriate ascertainment correction in analyses where constant-value features are absent, so that age estimates are not biased due to this absence.

In addition to variation in clock rate over the tree, BEASTling also provides support for variation in substitution rate across different linguistic features. If enabled, each feature is assigned its own substitution rate whose value is inferred during the analysis. These rates act as per-feature multipliers for the clock rate. Substitution rates are assigned a Gamma-distributed prior with a mean value of 1.0. The mean of all substitution rates is also constrained to be 1.0, so that the resulting rates are easily interpreted as rates relative to the average rate, e.g. a feature with rate 2.4 evolves at more than twice the average speed, while a rate of 0.13 is almost ten times slower than average. The Gamma distribution’s shape parameter, which determines the amount of variation in rate, is fitted to the data. Restricting the per-feature rates to have a mean of 1.0 still allows the shared clock rate to control the expected number of substitutions across the tree, given the inferred branch lengths. The need to support variation in substitution rate across features is very well established in linguistics. The assumption that no such variation exists was one of the strongest and earliest criticisms of the first formulations of glottochronology, and modern analyses have demonstrated the existence of considerable rate variation for both lexical [33] and typological [34, 35] data.

The probabilistic models within BEAST, used to compute the likelihood of data on a proposed tree, are composed of two main parts: equilibrium frequencies and a substitution model. BEASTling allows users to choose equilibrium frequencies to be uniform, which means that all values have equal equilibrium probabilities. However, by default the inference procedure assumes empirical frequencies, where each value’s equilibrium probability is proportional to its frequency in the dataset. For the substitution model which specifies the relative rates of transition between two states, several suitable for use with linguistic data are available.

The Lewis Mk model [20] is a generalised Jukes-Cantor model suitable for discrete features with an arbitrary fixed number of permitted states. Transitions are permitted from any state to any other, and all transitions are equally likely. This provides a simple and sensible default model for many types of linguistic data, including typological data from sources such as the World Atlas of Language Structures [19] (WALS) or South American Indigenous Language Structures [23] (SAILS). The Bayesian Stochastic Variable Selection (BSVS) model is a more complex model, similar to the well-known GTR model, in which some transitions may be more likely than others. BSVS extends GTR in allowing some transitions to be explicitly disallowed, which permits searching for directional preferences in the evolution of linguistic features. Precisely which transitions are disallowed is inferred during the MCMC analysis, and a prior distribution is placed on the number of allowed transitions.

The binary Covarion model [36] is defined for datasets where each feature has two permitted values, 0 and 1. The model permits a feature to transition between latent “fast” and “slow” states, which influence the rate at which transitions between 0 and 1 are permitted (transitions in either direction are equally probable). During the analysis, BEAST will estimate the rate at which features switch between the fast and slow states, and the difference in speed between the two states. This model has previously been applied to binary-encoded cognate data [2, 7]. This is its intended use in BEASTling, and the requisite recoding of cognate data can be applied automatically, along with the appropriate ascertainment correction. For example, a meaning slot with six attested cognate classes will be coded as six independent binary features, with languages typically receiving a 1 for one or two features and a 0 for all others. However, if BEASTling is provided with binary data (be it pre-binarised cognate data or typological data with two permissible values), it will recognise this and avoid a secondary binarisation.

In addition to substitution models for fitting trees to linguistic data, BEASTling also supports a spherical diffusion model [37] for fitting trees to location data. This model can be used to infer posterior locations for the homelands of language families, similar to a previous phylogeographic analysis of the Indo-European language family [7].

## Example analyses

To illustrate the sorts of analyses BEASTling is designed to facilitate, we present the results of two example analyses. Our intent is to concisely demonstrate the various abilities of the software, and these analyses should not be construed as serious attempts at historical linguistic scholarship. The BEASTling configuration files for both analyses are available as S1 and S2 Files in the Supporting Material. Further, the configuration files, data files and processing scripts required to replicate both of these example analyses are available in a GitHub repository at https://github.com/glottobank/BEASTling_paper/.

### Estimating Indo-European family tree from cognate data

Our first example is an inference of a phylogenetic tree for the Indo-European language family, using cognate data and the binary Covarion model. The dataset [38] (prepared by List [39] uses material from the “Tower of Babel” project [40]) and is comparatively small, containing 19 languages and 110 features, each of which corresponds to a word meaning. The datapoints are cognate class assignments, coded as integers. That is, two languages have the same integer for a given meaning if their words for that meaning are cognate. Known borrowings are indicated by negative values, i.e. a datapoint of -4 indicates that a language has borrowed a meaning from cognate class 4. Before running the analysis, we replace all known borrowings with question marks, so that they are treated by BEAST as missing data. Seven meanings in the dataset are automatically removed by BEASTling because they are constant for the 19 languages included, and thus cannot provide information about the tree topology (these meanings are *claw, name, new, salt, two, what* and *who*). Because the binary Covarion model was specified, BEASTling automatically reformats the cognate data for the 103 remaining meanings into binary form, resulting in 645 binary features. Because the languages in the datafile are identified by English names (“Dutch”, “Swedish”, “English”, etc.) and not ISO codes or Glottocodes, BEASTling cannot automatically impose monophyly constraints, so this feature is disabled. No calibration dates are provided, and rate variation across features is enabled.

**Fig 1:**
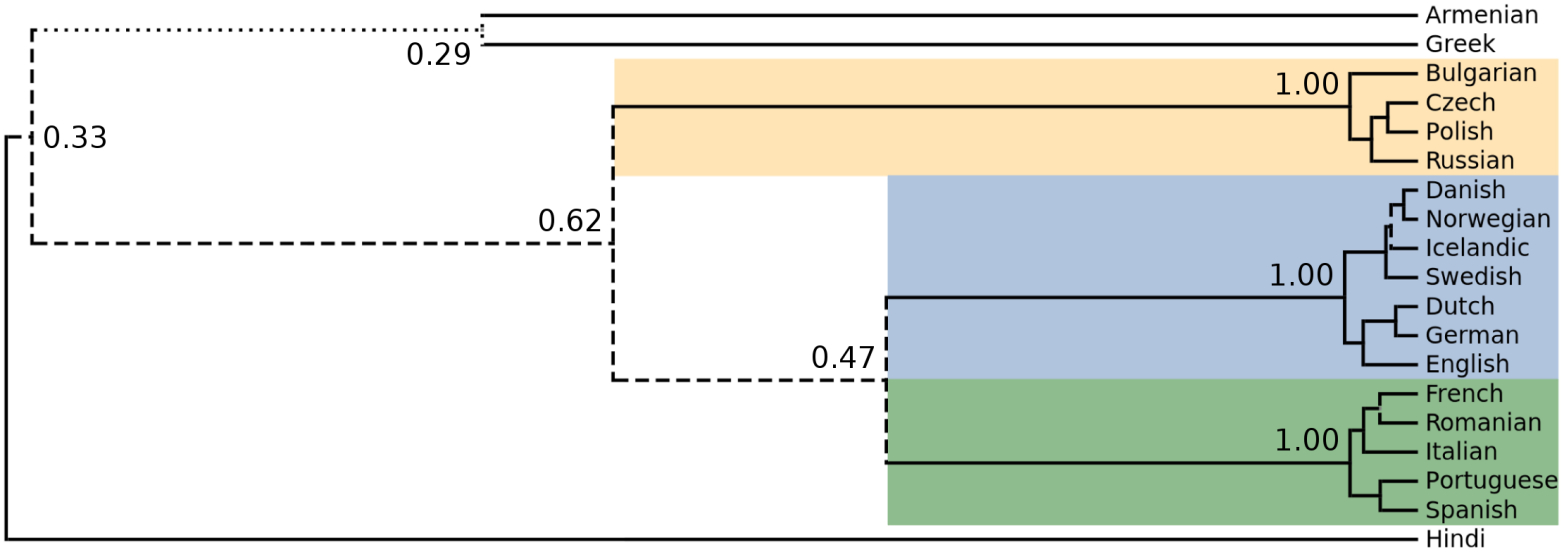
Maximum clade credibility tree for the Indo-European languages in our example analysis of Indo-European cognate data. The coloured blocks correspond to the correctly reconstructed subfamilies Slavic, Germanic and Romance. This tree is a summary of a posterior sample, and some aspects are more or less certain than others. Tree branches are solid if they subtend clades with posterior support exceeding 0.66 (common inside established subfamilies), dashed if support is between 0.33 and 0.66 and dotted if support is below 0.33 (which occurs only for the relationship between Armenian and Greek).

The maximum clade credibility tree produced by this analysis is shown in Fig 1. Note that despite the lack of monophyly constraints, the tree is in good agreement with conventional wisdom on Indo-European history. The Slavic, Germanic and Romance sub-families are all correctly positioned in their own clades. The Slavic clade is correctly divided into East, South and West Slavic, and the Germanic clade is correctly divided into North and West Germanic. The order in which Armenian, Greek and Hindi branch differs from previous analyses [1, 7], which may be at least partially due to the small number of languages in the dataset (note Hindi is the only representative of the Indo-Iranian subfamily). The close relationship between Romanian and French is also unexpected, and may be due to the influence of an erroneous cognate judgement in the dataset [41] as well as efforts at “purification” of Romanian [42]. It is important to understand that the tree shown in Fig 1 is one of a posterior sample of 10,000 trees, in particular the tree which best represents the relationships which are most strongly supported in the overall sample. Different parts of the tree topology are more or less strongly supported, and this is indicated graphically in the figure by the solidity of the branches. While the well-established Slavic, Germanic and Romance sub-families have posterior probabilities of 1.0, the more questionable Romanian-French clade has a posterior probability of 0.60 and the Armenian-Greek clade has a probability of just 0.29.

**Fig 2:**
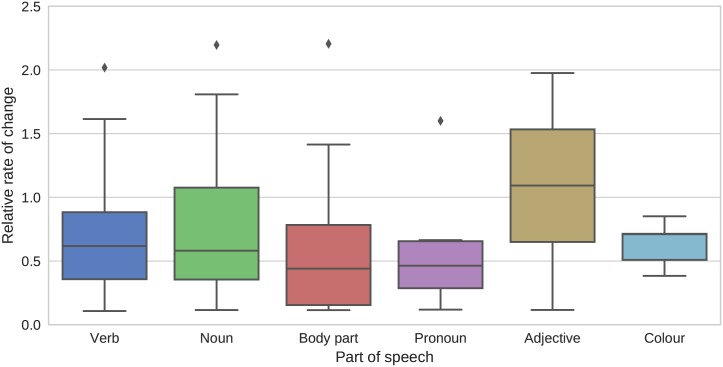
Distributions of relative substitution rates for different categories of meaning slot. Most categories have a median rate well below the average of 1.0, with long tails extending to faster than average rates. All colour terms have below average rates, as do all pronouns except for the outlier *that*.

In addition to a posterior sample of trees, the analysis logs posterior distributions over the relative substitution rate parameters for the 103 meaning slots. A considerable amount of rate variation is inferred, with the fastest meaning undergoing change 20 times faster than the slowest meaning. Table 1 shows the meanings with the ten highest and ten lowest rates, while Fig 2 shows how the distribution over rates varies across different parts of speech (see S1 Table for part of speech assignments). Verbs and nouns both have median rates well below the average of 1.0, with long tails toward higher rates. In contrast, adjectives have a median rate very close to average, with symmetric tails toward lower and higher rates. Words for body parts evolve somewhat more slowly than other nouns, and pronouns have a tight rate distribution with only a single outlier with an above average rate (the pronoun *that*), consistent with previous accounts of Indo-European pronouns showing little evidence of borrowing or grammaticalisation [43]. Similarly, colour terms are markedly more stable than other adjectives, with no colour terms having faster than average rates.

**Table 1:** Relative substitution rates of the ten slowest and fastest changing meaning slots in our example analysis of Indo-European cognate data.

Slowest		Fastest	
Feature	Rate	Feature	Rate
give	0.11	walk	1.61
tooth	0.11	heavy	1.63
sun	0.12	snake	1.68
full	0.12	big	1.76
I	0.12	short	1.76
star	0.12	woman	1.81
eye	0.12	many	1.98
ear	0.12	know	2.02
tongue	0.12	tail	2.20
heart	0.14	belly	2.21

Rates are relative to the average across all features, e.g. *tooth* evolves almost 10 times more slowly than average, while *know* evolves at just over twice the average rate. Note that many of the slowest meanings are body parts.

The rates obtained from the analysis allow us to order these meaning slots by stability. Previously, very similar word lists ordered by purported stability have been published by Swadesh [44], Starostin [45] and Pagel et al. [33]. Using electronic versions of these rankings available from the Concepticon project [46] we calculate the Spearman rank correlation coefficients between them and the ranking derived from our posterior mean substitution rates. The coefficients against the Swadesh and Starostin rankings are 0.46 and 0.45 respectively, and a slightly higher 0.57 against the mean of these two rankings (it is worth noting that both these authors based their conclusions on larger data sets than ours). Against Pagel et al.’s results, which are also based on Bayesian analysis of only Indo-European languages, albeit a different set of languages taken from a separate database, we get our highest correlation of 0.69. This kind of analysis paves the way for future studies of cross-linguistic variation in the relative stability of different meaning slots.

### Fitting substitution rates to WALS features using a fixed Austronesian tree

Our second example is an illustration of BEASTling’s ability estimate model parameters on the basis of a fixed tree, specified by the user. We use the maximum clade credibility tree from a 2009 Bayesian investigation of the Austronesian language family phylogeny by Gray et al. [2] as the fixed tree, and typological features from WALS [19] as the data. During the MCMC run, the tree topology and branch lengths are held fixed, while model parameters concerning the relative rates of different WALS features are sampled.

We label the leaves of the reference tree with ISO codes, and BEASTling automatically prunes the tree to include only those languages whose ISO codes are present in the WALS database. We configure BEASTling to exclude any features which have known values for less than 25% of languages. We also manually exclude 3 WALS features (IDs 95A, 96A and 97A) which are not features in their own right, but instead encode the relationship between other features (these feature exclusions are specified in the BEASTling configuration file and do not require editing of the data file). The final analysis involves 169 Austronesian languages and 25 WALS features (see S1 Appendix for a full discussion of the languages and features involved). A Lewis Mk model is specified for the data, with rate variation across features enabled. The inferred per-feature substitution rates are the subject of interest. Since the tree is fixed to a known value, BEASTling automatically disables tree logging to save disk space.

**Fig 3:**
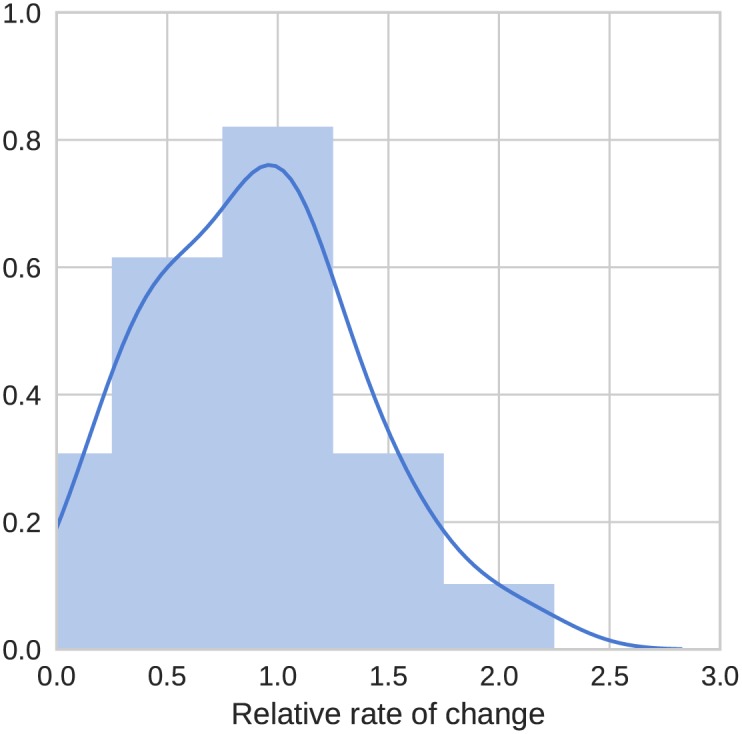
Histogram and kernel density estimate of relative substitution rates across the WALS features in our example analysis of Austronesian typological data.

The inferred rates of change of these typological features show a wider variation than the lexical rates of change in the Indo-European example above. The fastest changing feature has a rate around 27 times higher than the slowest changing feature. Table 2 shows the 10 slowest and fastest changing features, while Fig 3 shows a histogram and fitted distribution of the relative substitution rates across WALS features, which indicates that most features have a rate close to the average while below average rates are more common than above average rates. Many of the slowest features are categorised by WALS as word order features, consistent with a previous finding that these are some of the most stable structural features [47].

**Table 2:** Relative substitution rates of the ten slowest and fastest changing features in our example analysis of Austronesian typological data.

Feature	Rate
Slowest	
Order of Object and Verb	0.08
Order of Adposition and Noun Phrase	0.12
Order of Genitive and Noun	0.23
Position of Pronominal Possessive Affixes	0.27
Order of Subject and Verb	0.34
Order of Subject, Object and Verb	0.38
Preverbal Negative Morphemes	0.39
Order of Numeral and Noun	0.46
Position of Interrogative Phrases in Content Questions	0.47
Numeral Classifiers	0.50
Fastest	
Position of Tense-Aspect Affixes	1.19
Polar Questions	1.23
Position of Polar Question Particles	1.31
SVNegO Order	1.32
Weight Factors in Weight-Sensitive Stress Systems	1.41
Indefinite Articles	1.47
Definite Articles	1.60
Order of Degree Word and Adjective	1.63
Weight-Sensitive Stress	1.86
Fixed Stress Locations	2.14

Rates are relative to the average across all features. Note that many of the slowest features relate to word order.

## Availability and future directions

BEASTling is an open source project and full source code is available in a version control repository hosted by GitHub at https://github.com/lmaurits/BEASTling, under the terms of a 2-clause BSD license. BEASTling is also hosted at the Python Package Index (PyPI) and thus may be easily installed using standard Python packaging tools such as easy_install or pip. Searchable documentation, including a tutorial, is hosted by Read The Docs at https://beastling.readthedocs.org.

The authors intend to continually update BEASTling to support any new linguistically-relevant BEAST packages which may appear, and to keep model specifications in line with emerging consensuses on best practice. Contributions from the historical computational linguistics research community are welcomed.

## Supporting information

S1 File Indo-European example configuration file. (CONF)

S1 Table Categorisation of the meaning slots in the Indo-European example analysis used to display rate variation. (PDF)

S2 File Austronesian example configuration file. (CONF)

S1 Appendix Details of Austronesian example analysis language and feature sets. (PDF)
