# Supplementary material for: BEASTling: A software tool for linguistic phylogenetics using BEAST 2
Source: PLoS One. 2017 Aug 10;12(8):e0180908. doi: 10.1371/journal.pone.0180908 (PMC5552126; doi:10.1371/journal.pone.0180908)
Supplement: S1 Table — (PDF) [file pone.0180908.s002.pdf]

Table S2: **Categorisation of the meaning slots in the Indo-European example analysis used to display rate variation.** Note that the categories are disjoint: each meaning slot is assigned to one category only. So, the category of nouns should be thought of as “all those nouns which are not pronouns or body-parts”, and the category of adjectives should be thought of as “adjectives other than colours”. Note that a two meanings were excluded for the purposes of this figure: *not*, which is the only adverb in the data set, and *one*, which is the only numeral.

Meaning	Category	Meaning	Category	Meaning	Category	Meaning	Category
all	Pronoun	fat	Noun	louse	Noun	skin	Body part
ashes	Noun	feather	Noun	man	Noun	sleep	Verb
bark	Noun	fire	Noun	many	Adjective	small	Adjective
belly	Body part	fish	Noun	meat	Noun	smoke	Noun
big	Adjective	fly	Verb	moon	Noun	snake	Noun
bird	Noun	foot	Body part	mountain	Noun	stand	Verb
bite	Verb	full	Adjective	mouth	Body part	star	Noun
black	Colour	give	Verb	near	Adjective	stone	Noun
blood	Noun	good	Adjective	neck	Body part	sun	Noun
bone	Body part	green	Colour	night	Noun	swim	Verb
breast	Body part	hair	Body part	nose	Body part	tail	Noun
burn	Verb	hand	Body part	not	Excluded	that	Pronoun
cloud	Noun	head	Body part	one	Excluded	thin	Adjective
cold	Adjective	hear	Verb	person	Noun	this	Pronoun
come	Verb	heart	Body part	rain	Verb	thou	Pronoun
die	Verb	heavy	Adjective	red	Colour	tongue	Body part
dog	Noun	horn	Noun	road	Noun	tooth	Body part
drink	Verb	I	Pronoun	root	Noun	tree	Noun
dry	Adjective	kill	Verb	round	Adjective	walk	Verb
ear	Body part	knee	Body part	sand	Noun	warm	Adjective
earth	Noun	know	Verb	say	Verb	water	Noun
eat	Verb	leaf	Noun	see	Verb	we	Pronoun
egg	Noun	lie	Verb	seed	Noun	white	Colour
eye	Body part	liver	Body part	short	Adjective	wind	Noun
