# Supplementary material for: BEASTling: A software tool for linguistic phylogenetics using BEAST 2
Source: PLoS One. 2017 Aug 10;12(8):e0180908. doi: 10.1371/journal.pone.0180908 (PMC5552126; doi:10.1371/journal.pone.0180908)
Supplement: S1 Appendix — (PDF) [file pone.0180908.s004.pdf]

Details of Austronesian language and feature sets

The maximum clade credibility tree from Gray et al.'s 2009 publication contains 400 Austronesian languages, identified by written names such as "Sediq", "Bunan", "CentralAmis". Along with the tree, Simon Greenhill kindly provided us with a file mapping the 400 unique names to ISO codes, to facilitate attaching WALS data to the tree (the majority of languages in WALS have ISO codes assigned to them). However, some of the languages in the reference tree had no corresponding ISO code, and some ISO codes were used for multiple named languages. Similarly, many distinct languages in WALS are assigned the same ISO code. Achieving a unique mapping between languages in WALS and languages in the reference tree is therefore not a straightforward process. The final set of languages used for our example analysis, and their association with languges in WALS, was derived programmatically as follows.

First, all named languages in the 400 taxa tree which were not mapped to ISO codes were discarded. After this process, 395 of the original 400 languages remained in the tree.

Next, all ISO codes which mapped to multiple named Austronesian languages were identified. For each of these codes, we compared the list of all the names associated with that code in the Austronesian tree with the list of all the names of languages in WALS associated with that code. After converting all names to lowercase, if there was exactly one name which was common to both lists, then the WALS language with that name was associated with the language on the Austronesian tree associated with that name, and all other languages on the Austronesian tree associated with that ISO code were removed. If there was no such matching name, then *all* languages on the Austronesian tree corresponding to that ISO code were removed. After this process, 332 of the original 400 languages remained in the tree, with 332 unique ISO codes.

These 332 ISO codes map to a set of languages in WALS, and some ISO codes map to multiple languages. We resolved these duplicates in a similar manner to our resolution for duplicated ISO codes in the Austronesian tree. If an ISO code was associated with multiple WALS languages, but exactly one WALS language had a name which matched the (unique) name associated with that ISO code in the Austronesian tree, then that WALS language was retained and all others sharing its ISO code were removed. If no such match could be found, all languages with that ISO code were removed. After this process, 169 WALS languages are mapped to the tree via unique ISO codes.

The final languages' ISO codes and their names in the original Austronesian tree file are shown in Table 1. The WALS features for which these languages have data are shown in Table 2.

Table 1: **Languages in the Austronesian example analysis, including their ISO code and unique names as used by the Gray et. al. study and by WALS.**

ISO code	Gray et. al. name	WALS name	ISO code	Gray et. al. name	WALS name
agt	Agta	Agta (Central)	meu	Motu	Motu
akl	AklanonBisayan	Aklanon	mgl	Maleu	Maleu
akr	ArakiSouthwestSanto	Araki	mhs	BuruNamroleBay	Buru
alp	Alune	Alune	mhy	Maanyan	Ma'anyan
ami	CentralAmis	Amis	mhz	Mor	Mor
amk	AmbaiYapen	Ambai	min	Minangkabau	Minangkabau
ane	Canala	Xrc	mjk	Matukar	Matukar
aoz	Atoni	Timorese	mkj	Mokilese	Mokilese
apb	Saa	Sa'a	mlu	Toambaita	Toqabaqita
aty	AnejomAneityum	Anejom	mlv	Mwotlap	Mwotlap
ban	Bali	Balinese	mmn	Mamanwa	Mamanwa
bbc	TobaBatak	Batak (Toba)	mnv	Rennellese	Rennellese
bcl	BikolNagaCity	Bikol	mog	BolaangMongondow	Mongondow
bcm	Banoni	Banoni	mqy	Manggarai	Manggarai
bdl	Bajo	Bajau (Sama)	mri	Maori	Maori
bgz	BanggaiW_D	Banggai	mrn	ChekeHolo	Cheke Holo
bhp	Bima	Bima	mrq	Marquesan	Marquesan
bhq	Popalia	Tukang Besi	mrw	Maranao	Maranao
bhw	Numfor	Biak	mva	Manam	Manam
bkd	Binukid	Binukid	mxe	IfiraMeleMeleFila	Ifira-Mele
blw	Balangaw	Balangao	nal	Nalik	Nalik
bnq	Bantik	Bantik	nee	Nelemwa	Nelemwa
bps	SaranganiBlaan	Bilaan	nen	Nengone	Nengone
bug	BugineseSoppeng_D	Bugis	nfl	Aiwoo	Ayiwo
bzq	Buli	Buli (in Indonesia)	nia	Nias	Nias
cal	Carolinian	Carolinian	niu	Niue	Niuean
ceb	Cebuano	Cebuano	nkr	Nukuoro	Nukuoro
cha	Chamorro	Chamorro	nlg	Nggela	Gela
chk	Chuukese	Chuukese	nwi	TannaSouthwest	Tanna (Southwest)
cje	Chru	Chru	nxg	Ngadha	Ngad'a
cjm	PhanRangChamEasternCham	Cham (Eastern)	ojv	Luangiua	Luangiua
dbj	Idaan	Begak-Ida'an	pag	Pangasinan	Pangasinan
dgc	DumagatCasiguran	Dumagat (Casiguran)	pam	Kapampangan	Kapampangan
dhv	Dehu	Drehu	pau	Palauan	Palauan
dru	Rukai	Rukai (Tanan)	piv	VaeakauTaumako	Pileni
emb	Maloh	Embaloh	plh	Paulohi	Paulohi
emi	Mussau	Mussau	plt	MerinaMalagasy	Malagasy
erg	SyeErromangan	Erromangan	pma	PaameseSouth	Paamese
erk	SouthEfate	Efate (South)	pmf	Baree	Pamona
fos	Siraya	Siraya	pmt	Tuamotu	Tuamotuan
fud	FutunaEast	Futuna (East)	pon	Ponapean	Pohnpeian
gay	Gayo	Gayo	puw	Puluwatese	Puluwat
gfk	Patpatar	Patpatar	pwg	Gapapaiwa	Gapapaiwa
gil	Kiribati	Kiribati	pwn	Paiwan	Paiwan
gor	GorontaloHulondalo	Gorontalo	rap	RapanuiEasterIsland	Rapanui
haw	Hawaiian	Hawaiian	rej	RejangRejang	Rejang
hnn	Hanunoo	Hanunoo	rtm	Rotuman	Rotuman
hoa	HoavaNewGeorgia	Hoava	rug	Roviana	Roviana
huq	HainanCham	Tsat	sbe	Saliba	Saliba (in Papua New Guinea)
hvn	Savu	Sawu	sjr	Siar	Siar
iai	Iaai	Iaai	ski	Sika	Sika
iba	Iban	Iban	sku	SakaoPortOlry	Sakao
ibg	Ibanag	Ibanag	slp	Lamaholot	Lamaholot
ifb	IfugaoBatad	Ifugao (Batad)	slu	Selaru	Selaru
ilo	Ilokano	Ilocano	smo	Samoan	Samoan
ind	Indonesian	Indonesian	sov	PuloAnnan	Sonsorol-Tobi
irh	KasiraIrahutu	Irarutu	sps	Taiof	Taiof
isd	IsnegDibagatKabugaoIsneg	Isnag	ssf	Thao	Thao
jav	Javanese	Javanese	sun	Sunda	Sundanese
jmd	Yamdena	Yamdena	sxn	Sangir	Sangir
kei	KeiTanimbarKei	Kei	tah	TahitianModern	Tahitian
kge	Komering	Kayu Agung	tao	Yami	Yami
kij	Kilivila	Kilivila	tbc	Megiar	Takia
kje	Kisar	Kisar	tbl	TboliTagabili	Tboli
kkk	Kokota	Kokota	tgc	Tigak	Tigak
kos	Kusaie	Kosraean	tgl	Tagalog	Tagalog
kpg	Kapingamarangi	Kapingamarangi	tih	TimugonMurut	Timugon
ksd	Kuanua	Tolai	tio	Teop	Teop
ksx	Kedang	Kedang	tkl	Tokelau	Tokelauan
kwd	Kwaio	Kwaio	tkw	Buma	Buma
kxa	Kairiru	Kairiru	tnk	Kwamera	Kwamera
kzi	KelabitBario	Kelabit	tnl	Lenakel	Lenakel
lcm	TungagTungakLavongai	Tungak	tnt	Tontemboan	Tontemboan
leu	KaraWest	Kara (in Papua New Guinea)	ton	Tongan	Tongan
lex	Luang	Wetan	trv	Sediq	Seediq
ljp	Lampung	Lampung	tsg	TausugJolo_D	Tausug
llp	Nguna	Nguna	tsu	Tsou	Tsou
llu	Lau	Lau	tvk	AmbrymSouthEast	Southeast Ambrym
lml	Raga	Raga	twu	RotiTermanu_D	Roti
loj	Lou	Lou	txn	NgaiborSAru	Tarangan (West)
los	Loniu	Loniu	tzn	Tugun	Tugun
lti	Letinese	Leti	uur	Ura	Ura
mad	Madurese	Madurese	vnm	Neveei	Vinmavis
mah	Marshallese	Marshallese	wiv	Vitu	Bali-Vitu
mak	Makassar	Makassar	wlo	Wolio	Wolio
mbb	WBukidnonManobo	Manobo (Western Bukidnon)	wls	UveaEast	Wallisian
mbt	ManoboTigwaIglogsad_D	Kulamanen	woe	Woleaian	Woleaian
mek	Mekeo	Mekeo	wrp	Waropen	Waropen
mel	MelanauMukah	Melanau	xbr	Kambera	Kambera

Table 2: **WALS features included in the Austronesian example analysis.**

WALS feature ID	WALS feature Name
14A	Fixed Stress Locations
15A	Weight-Sensitive Stress
16A	Weight Factors in Weight-Sensitive Stress Systems
26A	Prefixing vs. Suffixing in Inflectional Morphology
27A	Reduplication
33A	Coding of Nominal Plurality
37A	Definite Articles
38A	Indefinite Articles
51A	Position of Case Affixes
55A	Numeral Classifiers
57A	Position of Pronominal Possessive Affixes
69A	Position of Tense-Aspect Affixes
81A	Order of Subject, Object and Verb
82A	Order of Subject and Verb
83A	Order of Object and Verb
85A	Order of Adposition and Noun Phrase
86A	Order of Genitive and Noun
87A	Order of Adjective and Noun
88A	Order of Demonstrative and Noun
89A	Order of Numeral and Noun
90A	Order of Relative Clause and Noun
90C	Postnominal relative clauses
91A	Order of Degree Word and Adjective
92A	Position of Polar Question Particles
93A	Position of Interrogative Phrases in Content Questions
94A	Order of Adverbial Subordinator and Clause
101A	Expression of Pronominal Subjects
112A	Negative Morphemes
116A	Polar Questions
143E	Preverbal Negative Morphemes
143A	Order of Negative Morpheme and Verb
143F	Postverbal Negative Morphemes
144K	SVONeg Order
144H	NegSVO Order
144D	The Position of Negative Morphemes in SVO Languages
144B	Position of negative words relative to beginning and end of clause and with respect to adjacency to verb
144I	SNegVO Order
144A	Position of Negative Word With Respect to Subject, Object, and Verb
144J	SVNegO Order
